# Ciliary protein ARL13b detected on RBCs as a potential indicative biomarker of vaso-occlusive crisis and disease severity in SCD: a retrospective pilot study

**DOI:** 10.1093/jscdis/yoag016

**Published:** 2026-03-13

**Authors:** Waseem Chauhan, Olivia Fernandez, Nirmish Shah, Amy Pan, Kevin R. Rarick, Ashraf M. Mohieldin, Surya M. Nauli, Sean Palecek, Ramani Ramchandran, Rahima Zennadi

**Affiliations:** 1 Department of Physiology, University of Tennessee Health Science Center, Memphis, TN, 38103, United States; 2 Duke Sickle Cell Comprehensive Care Unit, Duke University Hospital, Durham, NC, 27707, United States; 3 Division of Neonatology, Department of Pediatrics, Children’s Research Institute (CRI), Medical College of Wisconsin, Milwaukee, WI, 53226, United States; 4 Division of Critical Care, Department of Pediatrics, Children’s Research Institute (CRI), Medical College of Wisconsin, Milwaukee, WI, 53226, United States; 5 College of Graduate Studies, Master Program of Pharmaceutical Science, California North State University, Elk Grove, CA, 95757, United States; 6 Department of Medicine, University of California Irvine, Irvine, CA, 92697, United States; 7 Department of Chemical and Biological Engineering, College of Engineering, University of Wisconsin-Madison, Madison, WI, 53706, United States; 8 Department of Pediatrics, Division of Neonatology, Developmental Vascular Biology Program, Children’s Research Institute, Medical College of Wisconsin, Milwaukee, WI, 53226, United States

**Keywords:** red blood cells, ADP-ribosylation factor-like protein 13b, pain crisis, prognostic biomarker, disease severity predictor, sickle cell disease

## Abstract

**Objectives:**

Assessing SCD phenotype is challenging, hindering progress in care and quality of life. Pain crises are the hallmark of SCD, but no reliable prognostic biomarker exists due to its complex pathophysiology. ADP-ribosylation factor-like protein 13b (ARL13b), a small GTPase localized to primary cilia, is present on sickle RBCs and in plasma, therefore RBC-bound ARL13b may thus predict pain crises in SCD.

**Methods:**

Blood samples from SCD patients were analyzed for ARL13b on RBCs and soluble ARL13b (sARL13b). Retrospective chart reviews tracked hospitalizations for pain crises within 3 months before and after enrollment, hemolysis markers (hemoglobin, bilirubin, lactate dehydrogenase), and disease severity indicators (crisis frequency, acute chest syndrome, kidney disease, cerebrovascular accident). Correlations were calculated between ARL13b measures, pain crises, hemolysis, and severity.

**Results:**

In our pilot study, significant correlations existed between the percentage of RBCs carrying ARL13b measured on the day of enrollment, and the number of pain crisis experienced by the patients during the 3 months following enrollment (*r* = 0.3724, *P* = .0301), and clinical severity score (*r* = 0.5791, *P* = .0003). However, no correlation was found between the percentage of RBCs carrying ARL13b or sARL13b with the number of pain crisis experienced by the patients during the 3 months prior to enrollment, Hb, bilirubin, or LDH.

**Conclusion:**

In this exploratory study, ARL13b detected on sickle RBCs provides not only an objective prognostic biomarker of pain crisis, but also a potentially unbiased predictor of SCD severity.

## LAY SUMMARY

Our pilot study has identified a potential novel biomarker on red blood cells (RBCs) that could help assess the severity of SCD. We discovered that a protein called ARL13b, not previously known to be present on red blood cells, is found at elevated levels on the cells of individuals with SCD.

Our pilot study revealed a significant correlation: patients with a higher percentage of ARL13b-positive RBCs experienced more frequent painful vaso-occlusive crises and had a higher clinical severity score. This association was statistically strong, suggesting ARL13b levels may serve as an indicator of disease progression. The findings propose that measuring ARL13b could provide a valuable tool for stratifying patients by disease severity, potentially enabling more personalized treatment approaches and improved clinical management for this inherited blood disorder.

## GRAPHICAL ABSTRACT

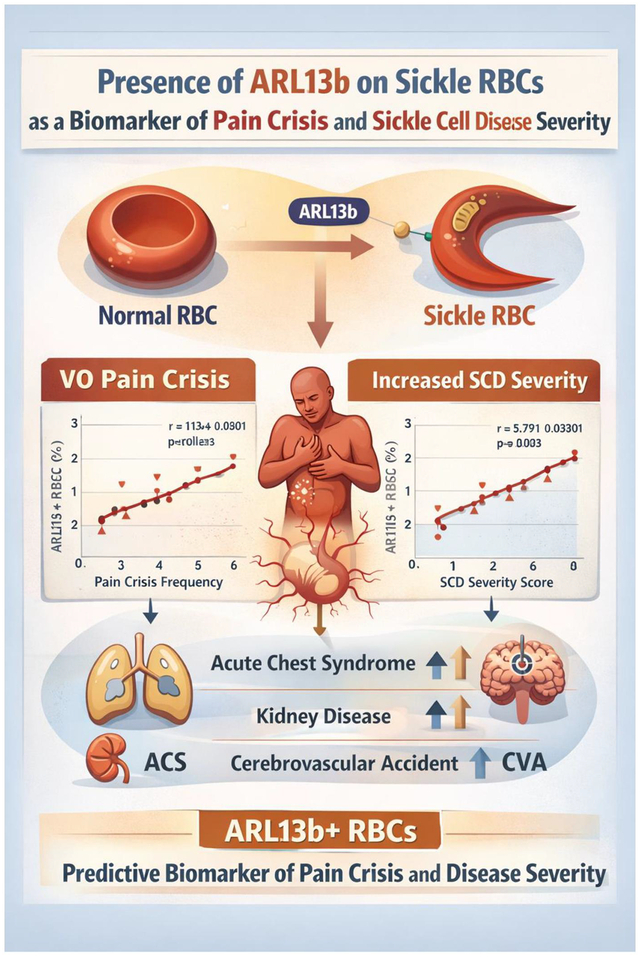

## INTRODUCTION

SCD is a genetic disorder that affects red blood cells (RBCs) which ultimately leads to multiorgan disease and early death. With an estimated 300 000 newborns affected globally each year—and projections indicating an increase to nearly 400 000 by 2050^1^—SCD represents a growing global health challenge. SCD is the result of a single point mutation in the *HBB* gene, in which the β-globin codon GAG is substituted with GTG, leads to a replacement of glutamic acid (Glu) to valine (Val) at position 6 of the β-globin chain. This alteration produces sickle hemoglobin (HbS). Under deoxygenated conditions, HbS polymerizes into rigid fibers, deforming RBCs into the characteristic sickle shape.^2^ RBCs are therefore, the core effector cells driving SCD pathophysiology. Their abnormal rigidity and enhanced adhesive properties make them the primary contributors to vaso-occlusion, as they interact with the vascular endothelium, leukocytes, and other blood cells to obstruct microvascular flow and precipitate vaso-occlusive crises (VOC) or “pain crisis.”^3–6^ The resulting ischemia can also lead to acute chest syndrome, stroke, multiorgan injury, and other severe or life-threatening complications.^2,7–10^

Beyond acute VOC events, SCD exhibits substantial phenotypic heterogeneity. Many patients demonstrate a hemolytic phenotype driven by chronic intravascular hemolysis, nitric oxide depletion, and vasomotor dysfunction, predisposing to pulmonary hypertension, leg ulcers, priapism, and ischemic stroke as well.^11^ Others manifest a vasculopathic phenotype marked by endothelial activation and proliferative vascular injury, contributing to cerebrovascular disease, renal dysfunction, and related vascular complications.^12^ Acute chest syndrome, an additional major cause of morbidity, often reflects intersecting inflammatory, vaso-occlusive, and hemolytic mechanisms.^13^ These overlapping phenotypes underscore the complex and multifactorial nature of SCD pathobiology and the genetic and epigenetic modifiers that shape individual disease trajectories.^9,14,15^

VOC is the most common clinical manifestation of SCD and the leading cause of hospitalization, yet its timing is unpredictable, which represents a substantial health burden for affected individuals. Improved ability to anticipate the timing and severity of VOC could substantially reduce morbidity by enabling earlier prevention.^16^ Despite interest in candidate biomarkers of endothelial activation—such as soluble intercellular adhesion molecule 1 (sICAM-1) and soluble vascular cell adhesion molecule 1 (sVCAM-1), which are typically found on vascular endothelial cells and have adhesive interactions with sickled RBCs,^17,18^ plasma thrombospondin 1, an activated platelet secretory molecule that mediates sickle cell adhesion, and vasoactive mediators including apelin and endothelin-1^7,16,19^—none have demonstrated reliable prognostic value for VOC due to insufficient correlation analyses or lack of validation across clinical studies.^20^ Moreover, pharmacologic trials targeting these pathways have not consistently reduced VOC frequency or management.^21^ Thus, identifying a mechanistically informed prognostic biomarker for VOC remains an urgent need.

Endothelial primary cilia, which extend into the vascular lumen, are hair-like, microtubule-based organelles that play a variety of roles in cardiovascular diseases.^22^ Cilia act as mechanoreceptors, detecting blood flow and adjusting intracellular responses to shear stress, which is the force exerted by blood flow on the surface of vascular endothelial cells.^22,23^ ARL13b is a cilia-enriched membrane protein and a molecular marker of ciliary integrity.^23^ During VOC, sickled RBCs adhere to the vascular endothelium, and obstruct blood flow.^24–28^ Our recent studies demonstrated that detachment of adherent sickle RBCs under physiological flow triggers endothelial deciliation, resulting in ciliary membrane shedding and transfer of the cilia-associated protein ARL13b onto RBC surfaces.^29^ Consistent with this mechanism, our prior work also showed that sickle RBCs carry more ARL13b than healthy RBCs and that plasma ARL13b levels are elevated in individuals with SCD.^29^ Because endothelial cilia are critical mechanosensory organelles, ARL13b detected on circulating RBCs or in plasma constitutes a biologically specific indicator of endothelial cilia loss and potentially an early form of vascular injury tightly linked to the initiation of vaso-occlusive pathology in SCD. We performed a pilot study to evaluate whether the ciliary protein ARL13b, detected on the surface of RBCs and in plasma from individuals with SCD, correlates with the occurrence of VOC, other clinical manifestations, or relevant laboratory parameters. The aim was to determine whether ARL13b levels are associated with disease activity in SCD, with a particular focus on their potential value as a candidate prognostic biomarker for future vaso-occlusive events and disease severity.

## MATERIALS AND METHODS

### Patient population

Following approval by Duke University Institutional Review Board and written informed consent, and the University of Tennessee Health Science Center, blood samples were collected from adult SCD patients aged 31–42 years (median 35 years; *n* = 30) and from age-matched healthy controls carrying hemoglobin A (Hb AA) (*n* = 27). Patients with SCD were excluded if they had been transfused in the past 3 months. Retrospective chart analysis was performed to track if the study participants were hospitalized for a VOC within the 3 months prior to enrollment and patients were prospectively followed to determine if VOC occurred within the 3 months after enrollment. We reviewed patient records to determine the occurrence of vaso-occlusive crises in the 3 months preceding and the 3 months following the date of blood collection, to increase the number of patients with at least one VOC event, while accommodating the wide inter-patient variability in VOC frequency. For this pilot study, VOC were defined as acute pain episodes requiring hospitalization, as documented in the medical record; outpatient-managed pain episodes were not included in the analysis. Additionally, records were reviewed to determine if the participant had a history of kidney disease, stroke, and acute chest syndrome, and the active medications that the participant was on. The participant’s hemoglobin, lactate dehydrogenase (LDH), and bilirubin were recorded at baseline.

### Blood collection and preparation

Human blood samples were collected into citrate tubes and were processed immediately. RBCs were separated centrifugally from plasma and buffy coat and washed extensively using phosphate-buffered saline (PBS). Packed RBCs were analyzed for leukocyte and platelet contaminations using an Automated Hematology Analyzer K-1000 (Sysmex Corporation, Kobe, Japan). Plasma samples were stored at −80°C.

### Flow cytometry

To determine the presence of the ciliary protein ARL13b on human RBCs, RBC purity was confirmed by light microscopy and by the absence of CD45^+^ leukocytes and CD41^+^ platelets in preliminary flow cytometry gates before staining. Single cell suspensions were washed 3× with PBS containing 1% bovine serum albumin (BSA) at 300*g* for 5 mins. Cells were then incubated for 30 minutes on ice with a with FITC-conjugated ARL13b antibody (Aviva, San Diego, CA). An FITC-conjugated isotype-matched IgG served as a negative control to assess nonspecific binding. After staining, RBCs were washed 3 additional times in PBS with 1% BSA. Samples were acquired on a FACScan flow cytometer (Becton Dickinson), and 100 000 events per sample were collected. During analysis, debris and non-cellular artifacts were excluded by forward-and side-scatter gating, and doublets were removed using FSC-H versus FSC-A plots to ensure single-cell analysis. Data were analyzed using FlowJo software (FlowJo, Ashland, OR).

### ELISA assay

Human sARL13b sandwich Elisa assay was performed to quantitate sARL13b in human plasma samples using human ARL13b Elisa kit (LS BIO, Shirley, MA) as recommended by the manufacturer.

### Statistical analysis

Categorical variables were compared by Fisher’s exact test while continuous variables were compared by *t* test or Welch’s *t* test. Some data were log transformed to meet parametric assumptions. Mann-Whitney-Wilcoxon or Kruskal-Wallis test with Dwass, Steel, Critchlow-Fligner Method for multiple comparison adjustment was used where parametric assumptions were not satisfied even after transformation. Spearman correlation coefficient (ρ) was calculated to examine the relationships between 2 continuous variables and scatter plot was generated to present the data. *P* < .05 was considered statistically significant. Data were analyzed using SAS version 9.4 (SAS Institute Inc., Cary, NC, United States).

## RESULTS

### Sickle cell patient population

Our study included a total of 30 patients with SCD, of which 14 were female (47%) (Table 1). The median age (31–42 years) at time of enrollment was 35 years (interquartile range [IQR] 31–42). The subtypes of patients with SCD were: 24 patients homozygous for hemoglobin S (Hb SS), 1 patient with Hb S beta zero thalassemia (Hb S/β^0^-Thal), and 5 patients with Hb SC (Table 1). All participants identified themselves as black or African American, and 67% of the SCD patients were on hydroxyurea (HU) (Table 1). RBCs isolated from blood collected from the 30 consented SCD patients were tested for ARL13b presence on the surface of these cells using flow cytometry assay, and isolated plasma samples were tested for soluble ARL13b (sARL13b) using ELISA assay. The SCD patients were tracked to determine if study participants were hospitalized for a VOC during the 3 months prior to enrollment and within the 3 months after enrollment (Table 1). The 30 enrolled SCD patients had baseline levels of hemoglobin (Hb), 23 of the patients had baseline LDH, and 22 of them had baseline bilirubin levels available at the day of enrollment (Table 1). Finally, all 30 patients enrolled had one or more clinical complication(s). Among the SCD patients tested, 17 patients (57%) have a history of pain crisis, 8 patients with a history of kidney disease (27%), 3 patients (10%) with a history of cerebrovascular accident, and 2 patients (6%) diagnosed with acute chest syndrome (Table 1).

### The ciliary protein ARL13b is detected on RBCs of sickle cell patients

**Figure 1.**
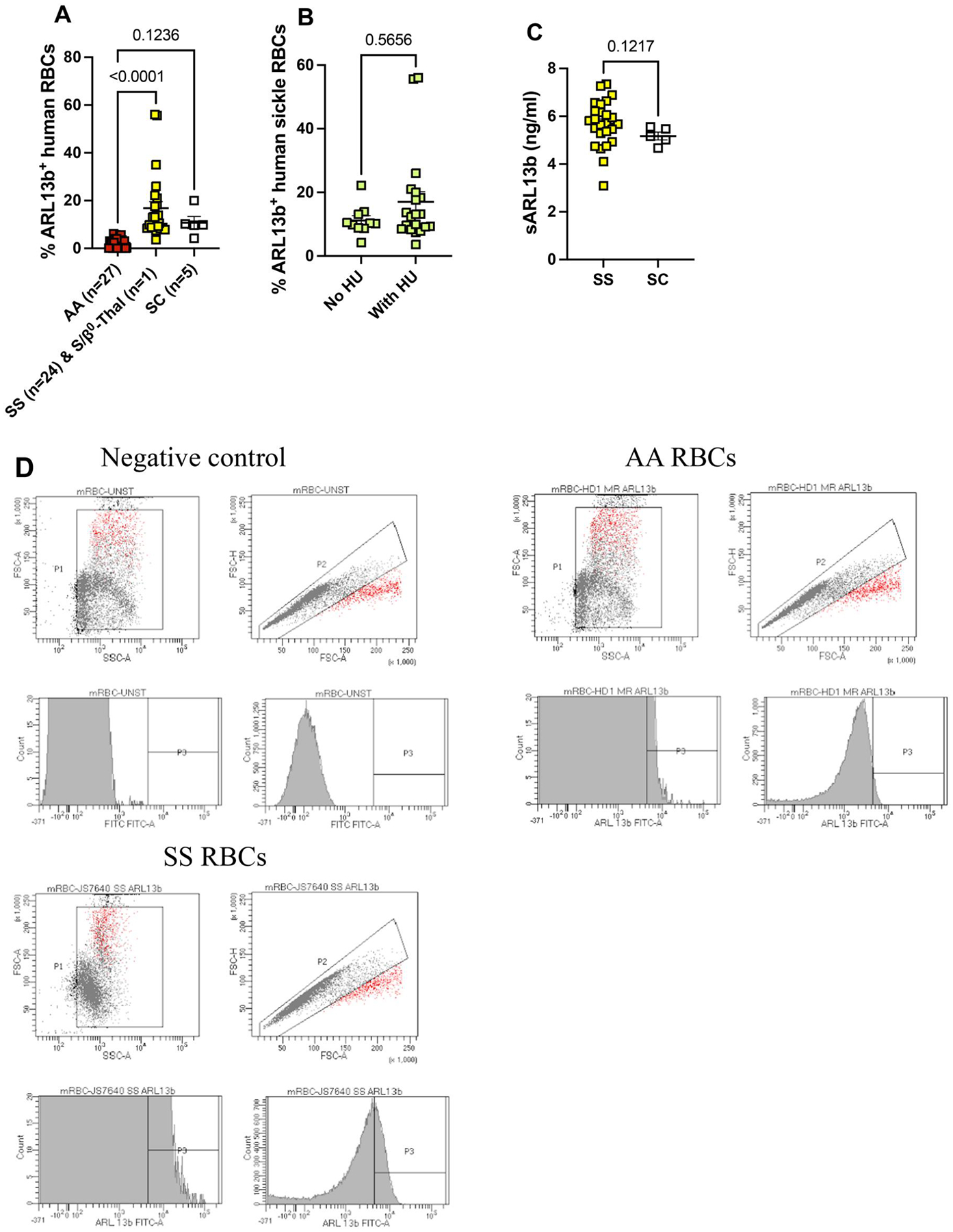
Presence of the ciliary protein ARL13b on human RBCs. (A) The presence of ARL13b on RBCs was higher among the combined Hb SS and Hb S/β^0^-Thal group (*n* = 25) than Hb AA volunteers (AA; *n* = 27), while Hb SC patients (SC; *n* = 5) were showing only modest presence for this ciliary marker on their surface. Error bars show SEM; *P* < .0001 vs. AA. (B) ARL13b-positive sickle RBCs in SCD patients with HU treatment. No significant difference in % ARL13b-positive sickle RBCs between SCD patients with HU treatment vs. without HU treatment. Error bars show SEM; *P* = .5656. (C) Plasma sARL13b. The levels of sARL13b in plasma of SS patients were equivalent to those detected in SC patients. Error bars show SEM; *P* = .1217. (D) A representative gating strategy for detection of ARL13b^+^ RBCs in healthy controls and SCD samples. Initial gating was performed using forward scatter (FSC) and side scatter (SSC) to identify the RBC population and exclude debris and non-cellular events. Doublets were removed by plotting FSC-Height (FSC-H) versus FSC-Area (FSC-A) to ensure single-cell analysis. Representative histograms show ARL13b-FITC fluorescence in RBCs from a healthy control (middle) and a SCD sample (right), with the isotype-matched FITC-IgG negative control (left). Quantification of ARL13b^+^ RBCs was performed using the isotype control to establish the positive fluorescence threshold. This gating strategy demonstrates clear separation between isotype background and ARL13b-positive events and was applied consistently across all samples.

We first confirmed our previous observation that the ciliary protein ARL13b can be detected on RBCs isolated from blood of the participants. Flow cytometry analysis revealed high presence of ARL13b on RBCs of sickle cell patients (Figure 1A). We observed significant differences in the percentages of ARL13b-positive (ARL13b^+^) human RBCs among healthy donors expressing Hb AA (*n* = 27), and SCD patients expressing severe disease genotypes, Hb SS (*n* = 24) and Hb S/β^0^-Thal (*n* = 1), whose data were combined based on their well-established comparable clinical severity and the standard practice of analyzing their genotypes together (*n* = 25; *P* < .0001). In contrast, no significant difference was detected between healthy donors and SCD patients expressing the milder Hb SC genotype (*n* = 5; *P* = .1236). Specifically, the percentage of ARL13b^+^ human RBCs was significantly lower in healthy donors (2.27% ± 0.41%) compared with the combined Hb SS and Hb S/β^0^-Thal group (16.84% ± 2.72%; adjusted *P* < .0001) (Figure 1A). However, no difference in ARL13b^+^ RBC levels was observed between patients receiving hydroxyurea (*n* = 20) and those not on hydroxyurea in this cohort (*n* = 10) (*P* = .5656; Figure 1B). Similarly, no difference was seen in the levels of sARL13b in plasma between SS patients and SC patients (*P* = .1217; Figure 1C).

**Figure 2.**
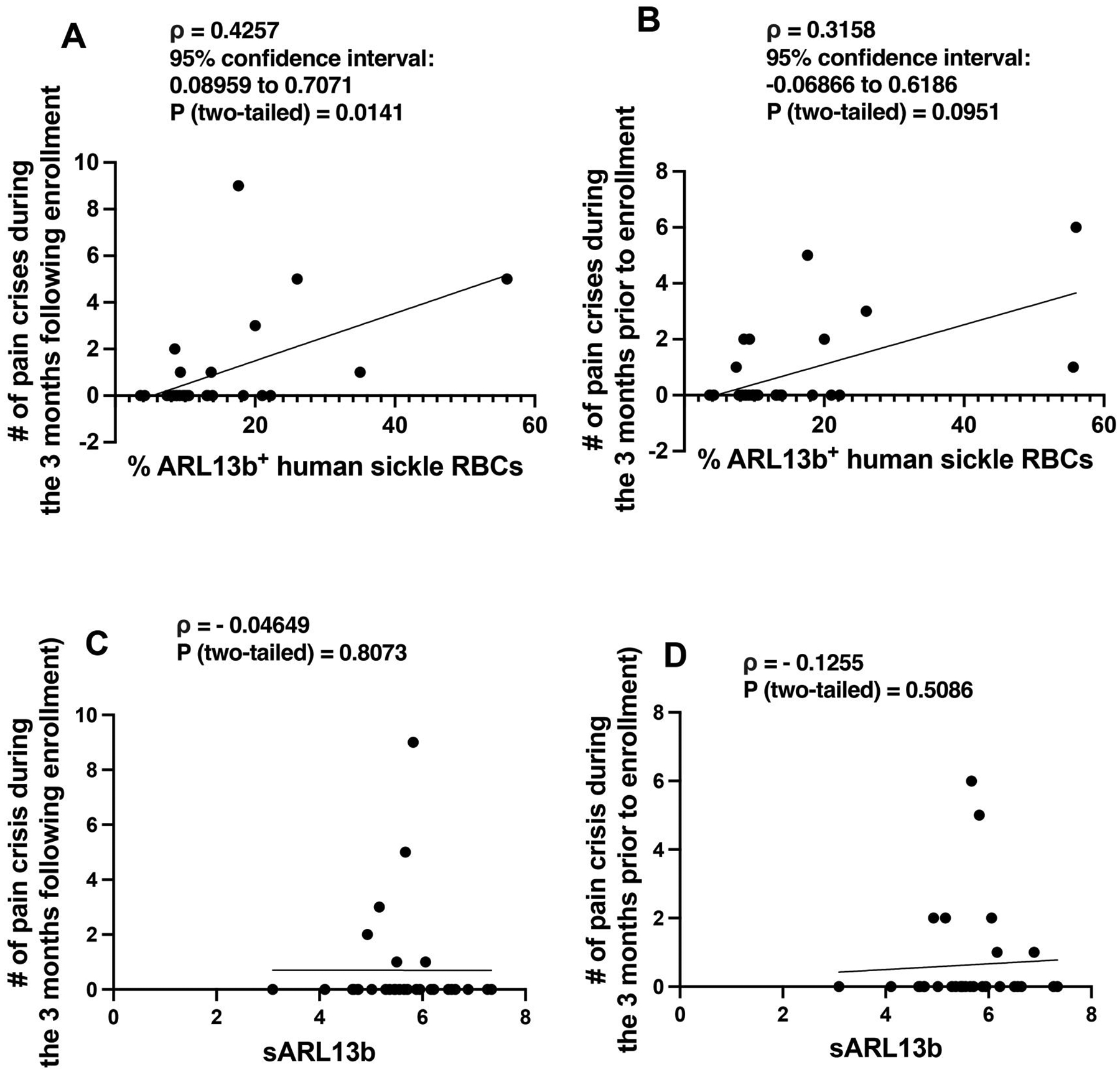
ARL13b-positive RBCs as a predictor biomarker for pain crisis in SCD. (A and B) Correlations exist between % ARL13b-bound RBCs from sickle cell patients (*n* = 30) and pain crisis during the 3 months following (ρ = 0.4257, *P* = .0141; A) but not prior to (ρ = 0.3158, *P* = .0951; B) enrollment. (C and D) No correlations between sARL13b from ρ sickle cell patients (*n* = 30) and pain crisis during the 3 months following (*P* = .8073; C) or prior to (*P* = .5086; D) enrollment.

To evaluate the clinical relevance of ARL13b presence on sickle RBCs, we assessed the association between the percentage of ARL13b^+^ RBCs at enrollment and pain crises reported by SCD participants during the 3 months preceding and following enrollment. The reasoning is that correlation with prior crises will establish whether ARL13b^+^ RBCs reflect recent disease activity, while correlation with subsequent crises may determine their predictive value for future crisis events. Demonstrating such associations would support ARL13b^+^ RBCs as a biomarker of vaso-occlusive risk, thereby linking a cellular phenotype to both recent clinical burden and short-term prognosis in SCD. Out of the 30 patients evaluated, 10 of them had VOC following enrollment. A positive correlation was detected between % ARL13b-bound RBCs and pain crisis experienced by sickle cell patients during the 3 months following enrollment (ρ = 0.4257, *P* = .0141; Figure 2A). However, when this same SCD patient cohort was examined for pain crisis during the 3 months prior to enrollment, no positive correlation was found between % ARL13b-bound RBCs and pain crisis (ρ = 0.3158, *P* = .0951; Figure 2B). In addition, no significant correlation was found between sARL13b and pain crisis during the 3 months prior to or following enrollment as well (Figure 2C and D). These data suggest that increased ARL13b presence on RBCs in SCD can be considered associated with impending pain crisis.

**Figure 3.**
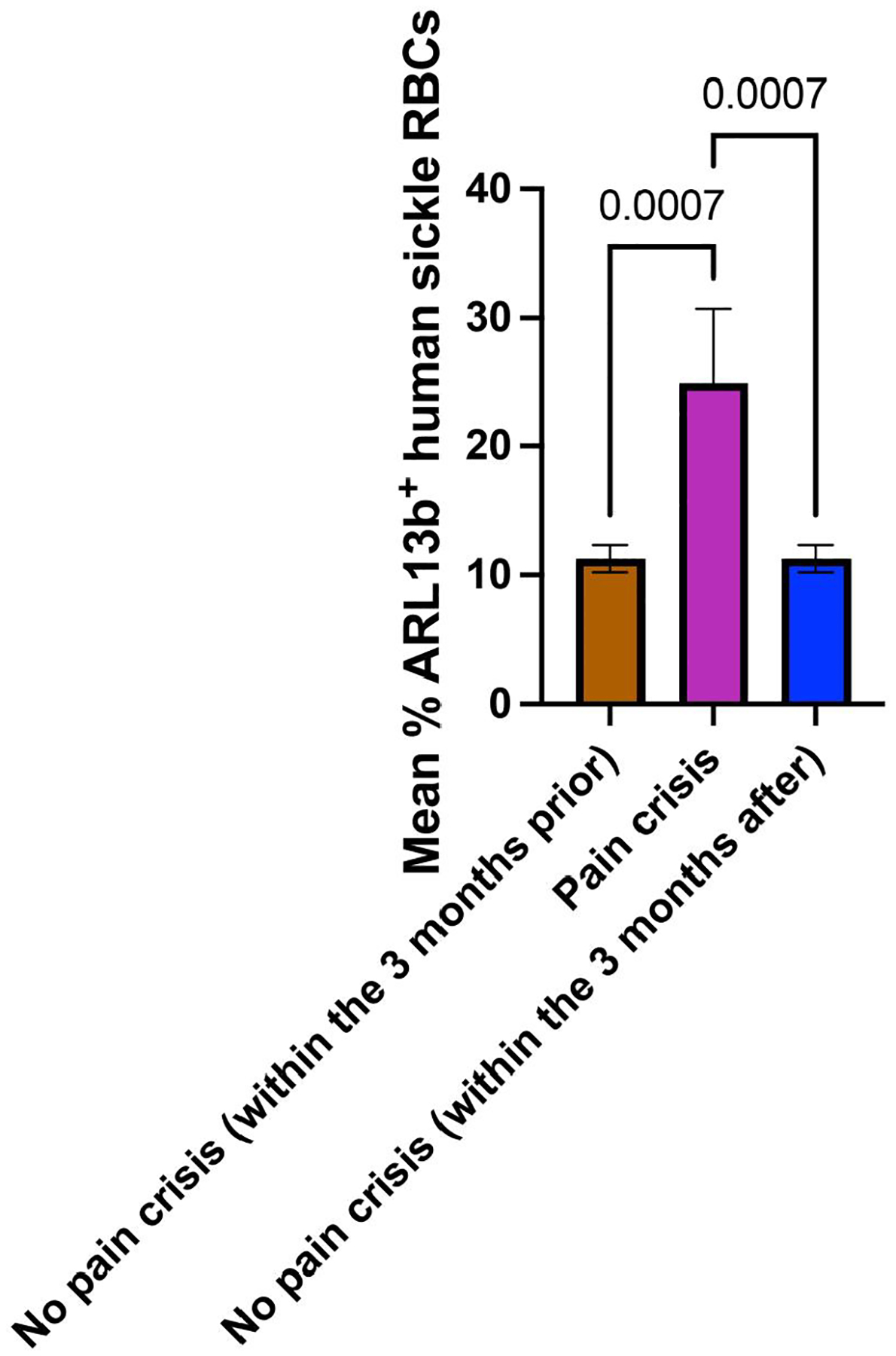
Association between mean percent ARL13b-bound RBCs and pain crisis. SCD patient participants who experienced pain crisis prior to and/or following enrollment were showing significantly elevated mean percentage of ARL13b-positive RBCs compared to patients with no pain crisis following or prior to enrollment. Error bars show SEM; *P* = .0007.

We subsequently determined the percentage and average of RBCs that were positive for ARL13b in participants who were actively in a pain crisis during the 3 months prior to and during the 3 months following enrollment and compared their values to participants who had no pain crises during those 2 timelines. This analysis was designed to test whether the proportion of ARL13b^+^ RBCs distinguishes participants who experienced vaso-occlusive crises in the 3 months before and after enrollment from those who remained crisis-free, thereby evaluating ARL13b as a potential biomarker of recent and short-term disease activity in SCD. Among the 30 evaluated SCD patients, we found that the mean percentage of RBCs positive for ARL13b was significantly higher (24.9.01 ± 5.8) for the 10 participants that were actively experienced pain crises compared to participants with no pain crises either during the 3 months following or prior to enrollment (11.29 ± 1.07%) (Figure 3).

### Presence of ARL13b on RBCs and correlation to anemia and hemolysis parameters

**Figure 4.**
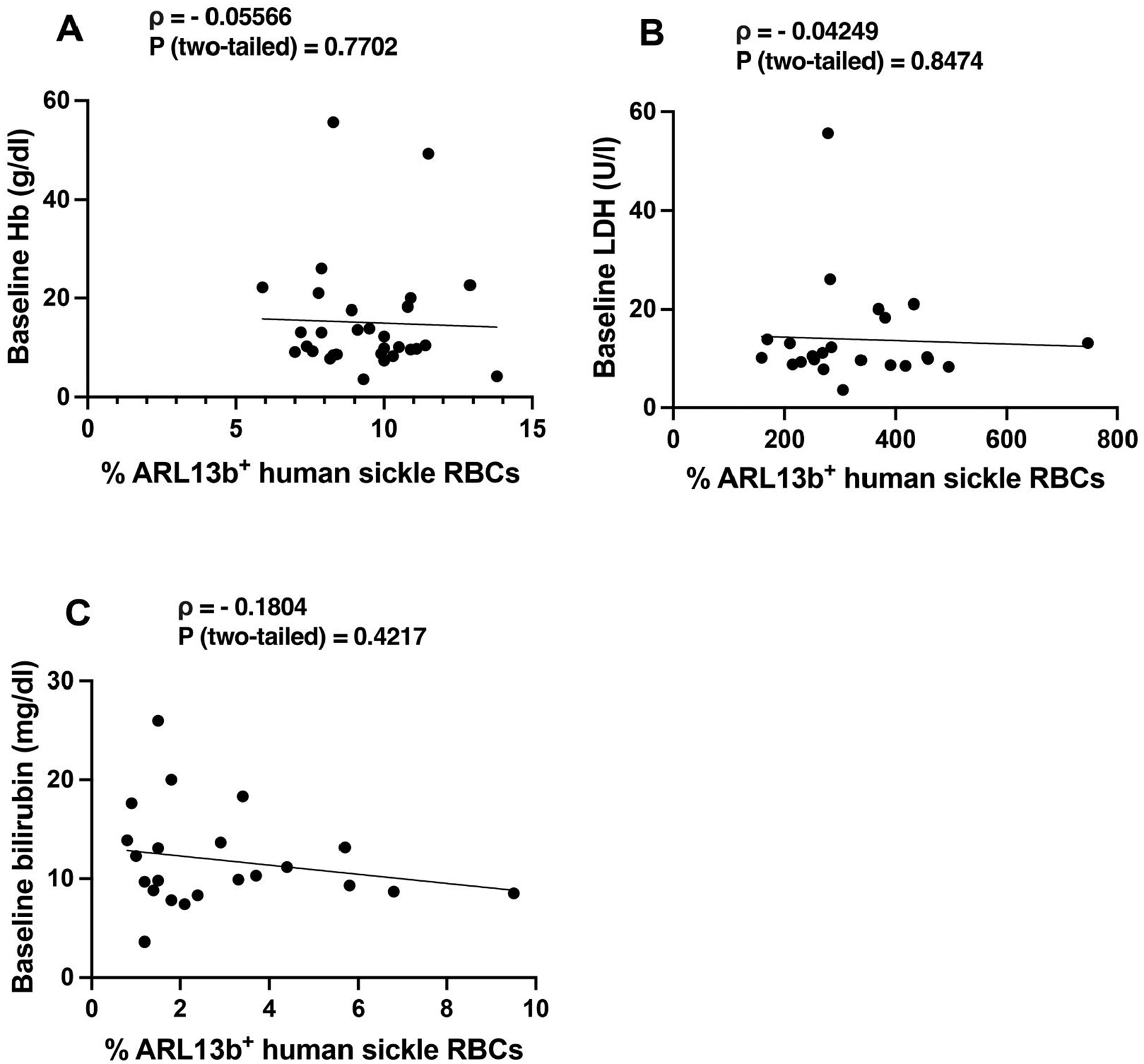
No significant correlation between ARL13b-positive RBCs and Hb, LDH or bilirubin. The levels of Hb (*n* = 30; A), LDH (*n* = 23; B), and bilirubin (*n* = 22; C) were not associated with % ARL13b-bound to RBCs.

We evaluated whether % ARL13b^+^ RBCs or plasma sARL13b correlate with hemoglobin, LDH, and bilirubin at baseline and during VOC hospitalization, to evaluate their potential as biomarkers of anemia and hemolysis in SCD. At both baseline and during VOC hospitalization (data not shown), hemoglobin, LDH, and bilirubin values showed no significant correlation with the proportion of ARL13b^+^ RBCs (Figure 4A–C) or with sARL13b concentrations (data not shown), suggesting that ARL13b-associated changes in RBCs occur independently of conventional hemolysis markers.

### Presence of ARL13b on RBCs and correlation with clinical complications and disease severity

**Figure 5.**
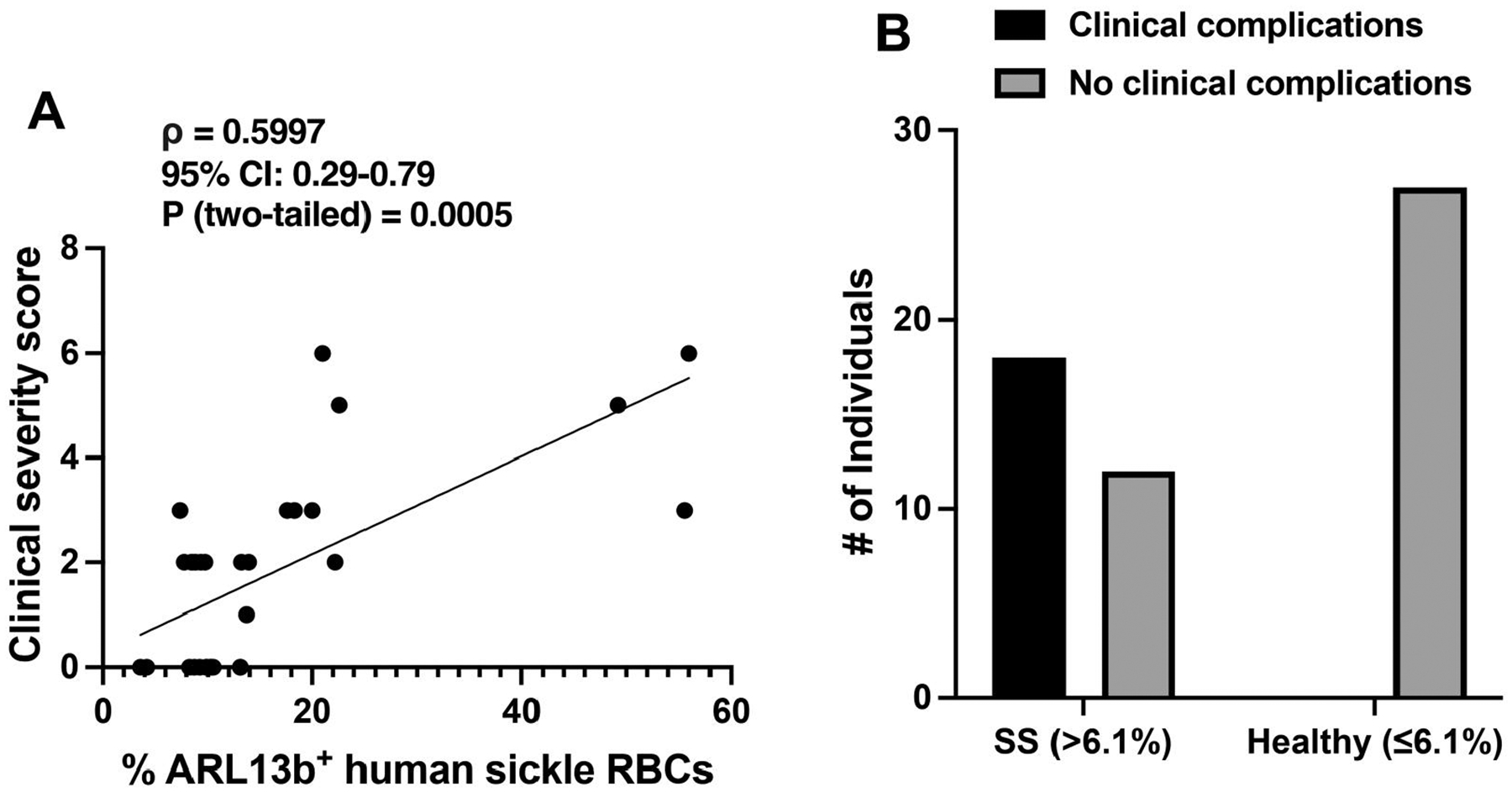
Increased presence of ARL13b on RBCs correlates with clinical severity and disease complications. (A) A positive correlation exists between % ARL13b-bound RBCs and clinical severity (*n* = 30; ρ = 0.5997; *P* = .0005). (B) The incidences of clinical complications were significantly higher among SCD patients who have percentages of ARL13b-positive RBCs higher than 6.1% (*P* < .0001).

To assess whether ARL13b deposited on RBCs reflects overall disease burden, we determined whether the degree of ARL13b bound to RBCs in SCD associates with clinical severity score. The clinical severity score was defined by the previously published Cincinnati Comprehensive Sickle Cell Center, which is calculated based on^30^ and shown in Table 1: number of hospitalization (for 5 or more hospitalization, the score is 3; for 2–4 hospitalization, the score is 2; and for 1 hospitalization, the score is 1); a history of kidney disease (the score is 2); a history of cerebrovascular accident (the score is 3); and a history of acute chest syndrome (the score is 2). A positive correlation existed between % ARL13b-bound RBCs and clinical severity score (*n* = 30; ρ = 0.5997, *P* = .0005; Figure 5A), supporting their role as a biomarker integrating cellular dysfunction with composite measures of clinical outcomes.

To establish a biologically meaningful cutoff point for ARL13b^+^ RBCs that distinguishes sickle cell patients with clinical complications from those without, we considered 6.1% ARL13b^+^ RBCs as a baseline, since healthy controls never exceeded this level (Figure 1). Because this cutoff was derived from healthy controls in the present cohort, future validation in larger and independent SCD populations will be necessary to confirm its generalizability and clinical relevance. Using 6.1% ARL13b^+^ RBCs as a cutoff based on healthy controls, this analysis tests whether SCD patients with clinical complications show elevated levels above this threshold, supporting its potential as a discriminator of complication risk. We found that a greater proportion of SCD patients with clinical complications (60%) exhibited ARL13b-positive RBCs exceeding 6.1% compared with those without complications (40%) (Figure 5B; *P* < .0001). These findings suggest that exceeding the 6.1% threshold of ARL13b^+^ RBCs, absent in healthy individuals, may be linked to the presence of clinical complications in SCD, indicating that ARL13b binding to RBCs could serve as a biomarker of disease severity and a stratification tool for patients at higher risk of adverse outcomes.

## DISCUSSION

In our pilot study, our findings provide evidence of increased ARL13b-positive RBCs as a candidate prognostic biomarker of vaso-occlusive pain crisis, as well as an indicator of clinical complications and disease severity in SCD. There is a critical need to identify biomarkers that correlate with VOC, the most common clinical manifestation of SCD and a major cause of organ damage and hospitalization. This exploratory study provides insight into a potential biomarker associated with VOC, as well as clinical complications and disease severity in SCD. Specifically, our findings indicate that increased ARL13b-positive RBCs above 6.1% may serve as a prognostic biomarker associated with pain crisis.

Our retrospective chart analysis captured whether participants were hospitalized for VOC within the 3 months prior to enrollment as well as after enrollment. We found that increased ARL13b-positive RBCs positively correlated with pain crisis experienced by SCD patients during the 3 months following enrollment. During VOC, sickled RBCs adhere to vascular endothelia,^31,32^ and our recent work demonstrated that under flow, adherent RBCs detach and trigger endothelial deciliation, resulting in attachment of the ciliary protein ARL13b to RBCs.^29^

As described, ARL13b is a protein located on cilia, which extend into the lumen of blood vessels. Cilia are mechanosensory organelles, housing many receptors including mechanoreceptors, detecting blood flow and adjusting intracellular responses to shear stress.^22,23,29^ Primary cilia play key roles in controlling the directional migration and barrier integrity of endothelial cells by modulating hsp27-dependent actin cytoskeletal organization.^33^ Complete loss of ARL13b causes disruption of cilia structure, protein trafficking, and sonic hedgehog signaling.^34^ Our previous studies have shown that endothelial deciliation occurs following disruption of vascular homeostasis, releasing ciliary fragments into circulation.^29^ Although circulating ARL13b has been proposed as a marker of vascular injury,^29,33^ we did not observe a correlation between sARL13b and pain crisis, potentially due to multiple biological sources of sARL13b, the vasculature and possibly hemolyzed RBCs positive for ARL13b, assay sensitivity, and the substantial inter-individual variability in VOC frequency in SCD.

In this exploratory study, we also observed no association between ARL13b-positive RBCs or sARL13b and laboratory measures of hemolysis (hemoglobin, bilirubin, LDH). This likely reflects the known variability in laboratory parameters across patients and across SCD genotypes as well. Nevertheless, the broader body of evidence suggests that endothelial dysfunction and vascular injury remain central to the pathophysiology of pain in SCD.^35^ Although correlation analysis was not performed, previous studies have demonstrated associations between endothelial activation markers, such as sE-selectin, and frequency of severe pain, supporting the relevance of vascular biomarkers in predicting clinical outcomes.^35^ Endothelial cells lining blood vessels are constantly damaged by cyclic ischemia/reperfusion or oxidative stress caused by RBC adhesion and VOC. Additionally, an imbalance between apelin (a vasodilator) and endothelin-1 (a vasoconstrictor) is associated with the vaso-occlusive phenotype.^16^ Future research will assess whether ARL13b-positive RBCs can predict other SCD complications and validate these findings in larger, more diverse cohorts.

Currently, there is no objective marker or physical exam finding to accurately diagnose or quantify pain in SCD, except for dactylitis in infants and young children,^36,37^ leading to verbal description of pain by a patient as the “gold standard” for understanding and assessing pain. Our pilot study highlights the potential role of ARL13b-positive RBCs in assessing frequent pain crises and the association of these positive cells with clinical manifestations and disease severity in this population. Endothelial dysfunction and its resulting damage are particularly relevant in SCD, contributing to the disease’s multifactorial pathology and long-term complications such as organ damage.^8,38^ Beyond ARL13b-positive RBCs, prediction models integrating clinical variables, molecular biomarkers, and gene expression profiling could further refine risk stratification and improve the identification of patients who may benefit from targeted interventions. Ultimately, combining clinical, laboratory, imaging, molecular, and genetic biomarkers may provide composite scores to predict specific complications, overall disease severity, and mortality.^15,39–43^

Several limitations should be acknowledged. This study was designed as a pilot investigation with a small cohort (*n* = 30), and thus the findings should be interpreted as preliminary. As a retrospective analysis, the timing of blood collection could not be standardized relative to endothelial injury or vaso-occlusive events, limiting insight into temporal changes in ARL13b-positive RBCs; future longitudinal sampling will be needed to capture dynamic shifts before, during, and after VOCs. In addition, documentation of VOC relied on medical records, which may underestimate crisis frequency, as many patients manage pain episodes at home without presenting for clinical care; incorporating patient-reported VOC data will be essential in future studies. Furthermore, sample availability was constrained by the requirement that blood be collected when patients were not experiencing a VOC, not receiving transfusions, and not showing signs of acute infection, combined with practical challenges in clinic attendance, as patients may miss routine appointments, manage crises at home, or have visits scheduled months apart. Finally, although a potential threshold for ARL13b-positive RBCs was identified, this cutoff requires validation in larger, prospective, and diverse cohorts to determine its robustness and clinical utility.

## CONCLUSION

In summary, this pilot study identifies elevated ARL13b-positive RBCs as a promising prognostic biomarker for vaso-occlusive crises and disease severity in SCD. While the findings are constrained by the small sample size and retrospective design, they underscore the biomarker’s potential clinical relevance in predicting pain crises and complications. Future research should focus on validating these results in larger, prospective cohorts, incorporating longitudinal sampling and patient-reported outcomes to better characterize the dynamic relationship between ARL13b-positive RBCs and VOC events. Ultimately, integrating ARL13b with other clinical and molecular markers may enhance risk stratification and guide targeted therapeutic strategies for SCD patients.

## Figures and Tables

**Table 1.** Sample demographic and clinical characteristics.

Demographic characteristics	
Ages (years)	
Median	35
Interquartile range (IQR)	31–42
Sex	*n* (%)
Female	14 (47)
Male	16 (53)
Ethnicity: African American	30 (100)
Clinical characteristics	
SCD genotype	
Hb SS or S*β*^0^	25 (83)
Hb SC	5 (17)
Treatment with hydroxyurea	*n* (%)
Yes	20 (67)
No	10 (33)

Number of pain crisis			
3 Months after enrollment		3 Months before enrollment	
Number	Individuals	Number	Individuals
0	22	0	22
1	3	1	2
2	1	2	3
3	1	3	1
≥4	3	≥4	2
History of pain crisis episodes		17 (57)	
Comorbidity		*n* (%)	
Kidney disease		8 (27)	
Cerebrovascular accident		3 (10)	
Acute chest syndrome		2 (6)	
Clinical severity and score			

Number of hospitalizations	Score
≥5	3
2–4	2
1	1
0	0
Comorbidity	Score
History of kidney disease	2
History of cerebrovascular accident	3
History of acute chest syndrome	2
Laboratory parameters (mean ± SEM)	
Hb (g/dL)	9.423 ± 0.3306 (*n* = 30)
LDH (U/L)	333.5 ±27.39 (*n* = 23)
Bilirubin (mg/dl)	2.936 ± 0.4824 (*n* = 22)

## Data Availability

All data are presented in the main manuscript.
